# Investigation of an Electrochemical Method for Separation of Copper, Indium, and Gallium from Pretreated CIGS Solar Cell Waste Materials

**DOI:** 10.1155/2015/494015

**Published:** 2015-08-12

**Authors:** Anna M. K. Gustafsson, Fredrik Björefors, Britt-Marie Steenari, Christian Ekberg

**Affiliations:** ^1^Industrial Materials Recycling, Chalmers University of Technology, Kemivägen 10, 412 96 Göteborg, Sweden; ^2^Department of Chemistry-Ångström Laboratory, Uppsala University, Box 538, 751 21 Uppsala, Sweden

## Abstract

Recycling of the semiconductor material copper indium gallium diselenide (CIGS) is important to ensure a future supply of indium and gallium, which are relatively rare and therefore expensive elements. As a continuation of our previous work, where we recycled high purity selenium from CIGS waste materials, we now show that copper and indium can be recycled by electrodeposition from hydrochloric acid solutions of dissolved selenium-depleted material. Suitable potentials for the reduction of copper and indium were determined to be −0.5 V and −0.9 V (versus the Ag/AgCl reference electrode), respectively, using cyclic voltammetry. Electrodeposition of first copper and then indium from a solution containing the dissolved residue from the selenium separation and ammonium chloride in 1 M HCl gave a copper yield of 100.1 ± 0.5% and an indium yield of 98.1 ± 2.5%. The separated copper and indium fractions contained no significant contamination of the other elements. Gallium remained in solution together with a small amount of indium after the separation of copper and indium and has to be recovered by an alternative method since electrowinning from the chloride-rich acid solution was not effective.

## 1. Introduction

The semiconductive material copper indium gallium diselenide (CIGS) is used in high efficiency thin film solar cells. To ensure a future supply of the rare and valuable metals indium and gallium the material needs to be recycled [1, 2]. Recycling of CIGS waste materials often involves hydrometallurgical treatment that includes dissolution, precipitation, and solvent extraction for separation of the elements [3–7]. Electrodeposition has been used for the recovery of indium after separation [5], and Drinkard Jr. et al. showed that electrodeposition can also be used for separating copper and selenium from indium [3]. To our knowledge, however, no one has used electrodeposition to separate copper, indium, and gallium from each other.

The standard reduction potentials (*E* ^0^) for the reduction of copper(II), indium(III), and gallium(III) according to reactions (1), (2), and (3) are reported to be 0.34, −0.338, and −0.529 V versus the standard hydrogen electrode, respectively [8]:

(1) Cu2+aq+2e−⟶Cus

(2) In3+aq+3e−⟶Ins

(3) Ga3+aq+3e−⟶Gas

The differences in standard reduction potentials indicate that separation of copper from indium and gallium should be possible to be done by electrodeposition. On the other hand, separation of indium from gallium could be more difficult, since the standard reduction potentials are relatively similar. However, the actual potential needed for reduction usually deviates from the standard potential due to several different factors: the deviation from the standard state [9], the complexation of the desired element in solution [10], and the activation overpotential needed to overcome the potential barrier at the interface between the electrode and the solution [11]. A literature study showed that electrodeposition of gallium from aqueous solutions requires large overpotentials [12–14] and that high hydrochloric acid concentrations inhibit the electrodeposition of gallium [15]. It has also been found that electrodeposition of gallium from an aqueous solution containing both indium and gallium, using a controlled current, begins only after most of the indium has been removed from the solution [14]. In addition, it has been shown that indium and gallium can be separated by electrodeposition of indium from solutions with high sulfuric acid concentrations [16].

We have in a previous study shown that selenium can be separated from the other elements via oxidation at elevated temperatures [17]. The residue from the proposed selenium separation process contains oxides of copper, indium, and gallium and the next step in the development of a recycling process for the CIGS material is to find an effective method for separating these elements in pure form. Unpublished work has shown that the selenium-depleted material can be dissolved by two different methods. The first method results in an approximately 1 M hydrochloric acid solution that contains the elements. The second method gives a solution of 1 M hydrochloric acid that contains the elements and ammonium chloride.

The goal of the present study was to investigate if copper, indium, and gallium in the two solutions could be separated by consecutive electrodeposition, of firstly copper, then indium, and finally gallium. The reduction potential for each element in the selected systems was first investigated by cyclic voltammetry on synthetic solutions prepared by dissolving the chloride salts in 1 M hydrochloric acid. The separation of the elements was then tested using a synthetic solution of the elements in 1 M hydrochloric acid before the separation was done from the solutions containing the dissolved CIGS waste material.

## 2. Materials and Methods

### 2.1. Cyclic Voltammetry

The redox behavior of copper(II), indium(III), and gallium(III) in chloride solutions was studied with cyclic voltammetry. The experiments were performed in a three-electrode cell connected to a galvanostat (VersaSTAT 3, Princeton Applied Research) at a scan rate of 100 mV/s. The three electrodes used were a working electrode of glassy carbon (MF-2012, BASi), a platinum wire counter electrode (MW-1032, BASi), and a silver/silver chloride reference electrode (MF-2052, BASi). The working electrode was polished with alumina polish (CF-1050, BASi) between experiments to remove residual deposits on the surface.

First synthetic metal solutions, containing 0.05 M copper(II), indium(III), or gallium(III), were prepared by dissolving copper(II) chloride (99.999%, Sigma-Aldrich), indium(III) chloride (99.999%, Sigma-Aldrich), or gallium(III) chloride (99.999%, Sigma-Aldrich) in 1 M hydrochloric acid. The acid was prepared by dilution of hydrochloric acid (puriss, 37%, Sigma-Aldrich) in ultrapure water obtained from a Milli-Q system (>18 MΩ·cm, Millipore Advantage A10). The electrolyte was also analyzed to determine the background signal and solutions containing 0.05 M of indium and gallium, or all three elements, were tested to investigate if the redox behavior of the elements were influenced by each other. Finally the solutions of dissolved selenium-depleted CIGS waste material was analyzed to determine possible differences in behavior compared to the synthetic solutions.

### 2.2. Electrochemical Separation of Copper, Indium, and Gallium from a Synthetic Chloride Solution

To investigate if selective electrodeposition could be used to separate copper, indium, and gallium the separation was first performed using a synthetic solution containing 0.25 M of copper(II), indium(III), and gallium(III) in 1 M hydrochloric acid (Solution 1). The solution was prepared from the metal chlorides in the same way as the solutions used for the cyclic voltammetry study. The separation was done first by the electrodeposition of copper and then indium from the solution at suitable reduction potentials.

The experimental setup for the separation study was the same as that for the cyclic voltammetry study, with the exception that the working electrode was changed to a glassy carbon rod (GC 20SS, Tokai Carbon Co.) with a diameter of 3 mm, while the counter electrode was a thick (*Ø* 8 mm) glassy carbon rod. During the experiment the working electrode was submerged 10 mm into the solution. For each experiment 50 mL of solution was used and stirring at 500 rpm facilitated a homogeneous concentration of the metals. Firstly copper was separated from the solutions by applying a constant potential of −0.5 V (versus Ag/AgCl) for 8 h. During the experiments the current was recorded and samples (10 *μ*L) were taken from the solution after 0.25, 1, 2, 4, 6, and 8 h. The samples were diluted in 0.1 M nitric acid made from Suprapur nitric acid (65%, Merck) and Milli-Q water and were analyzed with ICP-OES (Thermo Scientific iCAP 6500). After copper separation indium was separated from the solutions using a constant potential of −0.9 V (versus Ag/AgCl) for 8 h. The current was recorded and samples of the solution were taken and analyzed in the same way as that for the copper separation. The copper and indium deposits were weighed before they were dissolved in concentrated nitric acid (puriss, 69%, Sigma-Aldrich) and analyzed with ICP-OES. All experiments were performed in triplicate to evaluate uncertainty.

### 2.3. Electrochemical Separation of Copper, Indium, and Gallium from Solutions of Dissolved Selenium-Depleted CIGS Waste Material

After the experiments using the synthetic solution the same procedure was tested on solutions of dissolved selenium-depleted CIGS waste material to determine if the separation method could be used for the recycling of copper, indium, and gallium. Prior to the dissolution XRD showed that the residue from the selenium separation consisted of In_2_O_3_, CuO, CuInGaO_4_, and Cu_2_In_2_O_5_, and ICP-OES analysis showed that the material contained 26.2 ± 1.3 wt% copper, 43.9 ± 0.7 wt% indium, 6.4 ± 0.2 wt% gallium, 0.3 ± 0.1 wt% selenium, and 23.2 ± 1.1 wt% oxygen. Two solutions were prepared from the selenium-depleted CIGS material, using different dissolution methods.

#### 2.3.1. Dissolution of Selenium-Depleted CIGS Material in 3 M Hydrochloric Acid and Hydrogen Peroxide

The first solution containing real CIGS waste material (Solution 2) was prepared by dissolving 1.5 g of the residue from the selenium separation in 46 mL 3 M hydrochloric acid at 75°C and adding 1 mL/h of hydrogen peroxide (30%, Sigma-Aldrich) over 4 h. The solution was stirred at 500 rpm during the dissolution. After the dissolution the leachate was diluted with Milli-Q water to 50 mL. ICP-OES analysis showed that the metal concentrations in the resulting solution were 0.24 ± 0.01 M copper, 0.21 ± 0.01 M indium, 0.061 ± 0.002 M gallium, and 0.005 ± 0.001 M selenium. The pH of the solution was determined by acid/base titration to be roughly 0.

#### 2.3.2. Dissolution of Chlorinated, Selenium-Depleted CIGS Material in 1 M HCl

The second solution containing dissolved CIGS waste material (Solution 3) was prepared from a selenium separation residue that had first been chlorinated to increase solubility. The chlorination procedure has been described in detail elsewhere [18]. In this case oxidized material was mixed with ammonium chloride at a weight ratio of 1 : 1.5 (oxidized material : ammonium chloride) and heated in a furnace at 650°C for 2 h before the chlorinated product was collected. 8 g of chlorinated product was dissolved in 50 mL 1 M hydrochloric acid. To remove small amounts of precipitated selenium residues the solution was filtered prior to ICP-OES analysis. The metal concentrations in the solution were 0.24 ± 0.01 M copper, 0.22 ± 0.01 M indium, and 0.066 ± 0.001 M gallium. The selenium concentration was below the detection limit of the ICP-OES. Also in this case the pH of the solution was close to 0.

## 3. Results and Discussion

### 3.1. Cyclic Voltammetry

**Figure 1:**
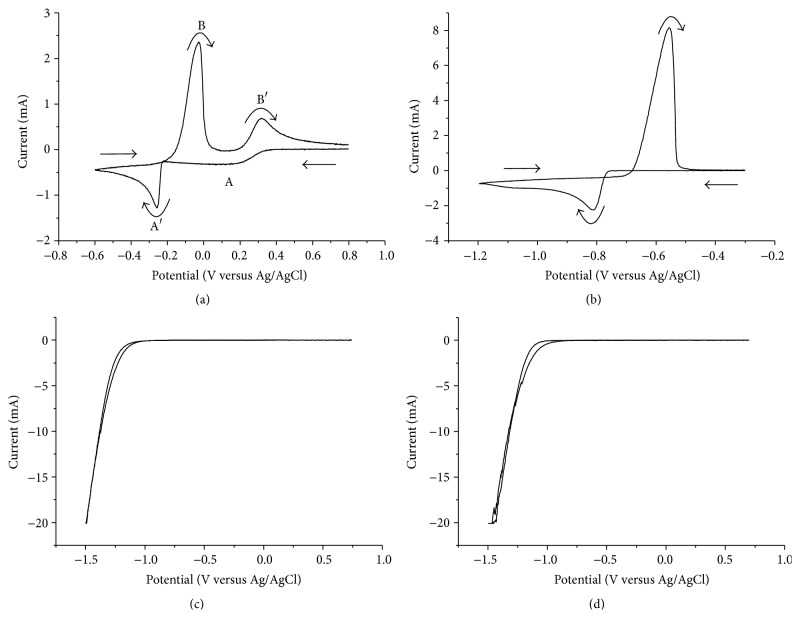
Voltammogram for 0.05 M (a) copper(II), (b) indium(III), and (c) gallium(III) in an electrolyte of 1 M HCl (d). The solutions were analyzed using a scan rate of 100 mV/s.

The results from the cyclic voltammetry experiments are presented in Figure 1.

The cyclic voltammogram recorded for copper is shown in Figure 1(a). In the figure two cathodic voltammetric waves (A and A′), at approximately 0.25 and −0.25 V, can be seen. Napp and coworkers have shown that copper(II) is reduced to copper(0) via the intermediate copper(I) in the chloride media [19] and the formation of CuCl, via the following reaction on the electrode surface has been confirmed [20]:

(4) $$\begin{array}{c} {Cu}^{2+}\left(\;aq\;\right)+{Cl}^{-}\left(\;aq\;\right)+e^{-}⟶CuCl\left(\;s\;\right) \end{array}$$

The reduction to copper(I) explains the first reduction wave (A) in the cyclic voltammogram. At more negative potentials copper(I) is further reduced to copper(0), according to the following reaction [20]:

(5) $$\begin{array}{c} CuCl\left(\;s\;\right)+e^{-}⟶Cu\left(\;s\;\right)+{Cl}^{-}\left(\;aq\;\right) \end{array}$$

The reduction of copper(I) takes place in parallel with the direct reduction of copper(II) to copper(0), according to reaction (1) [20, 21]. The combined result is the second, larger reduction wave (A′). The shift in the potential needed for copper reduction to more negative values compared to the standard reduction potential is due to the factors discussed in the theory section. As an example, the complexes formed in chloride solution are more stable than the hydrated complexes in a pure water solution. In the reversed scan copper(0) on the electrode is oxidized giving rise to the first metal stripping peak (B) at 0 V. Depending on the chloride concentration copper(0) is either oxidized to copper(I) or directly to copper(II) [22]. Above 1 M chloride copper(I) is the dominating species and this gives rise to the second metal stripping peak (B′), at 0.3 V, as copper(I) is oxidized to copper(II).

For indium(III) (Figure 1(b)) the cathodic voltammetric wave at −0.8 V that was seen in the cathodic scan was attributed to the reduction of indium(III) by reaction (2). In the reverse scan the oxidation of the formed indium metal gave one anodic wave at −0.57 V. The difference in potential between the reduction of copper(II) and indium(III) indicated that a separation of copper from indium could be achieved by electrodeposition of copper.

For gallium(III) (Figure 1(c)) only reduction of water at potentials below −1 V was observed, even though the potential window was extended to −1.5 V. The result was the same as for the electrolyte without any metal species (Figure 1(d)). It is clear that the actual reduction potential for gallium requires large overpotentials and that these results agree well with those reported by other authors sited in the theory section. This means that it is difficult, or even impossible, to electrowin gallium from this solution and that the separation of indium from gallium by electrodeposition should be easily achieved.

**Figure 2:**
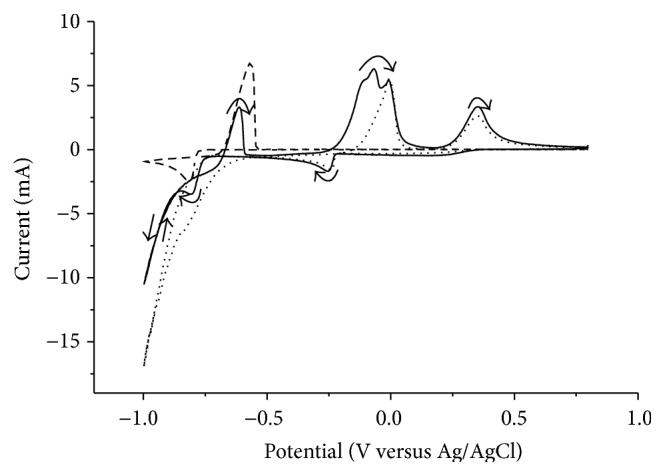
Voltammogram of a solution containing copper(II), indium(III), and gallium(III) (solid line) compared to a solution containing only copper(II) (dotted line) or indium(III) (dashed line) at a scan rate of 100 mV/s.

To investigate if the reduction potential of copper was influenced by indium and gallium a solution containing all three elements was analyzed. The results presented in Figure 2 show that the reduction potentials for copper(II) and indium(III) were the same in the solutions containing all three elements as in the solutions containing only copper or indium. In the reverse scan the indium oxidation wave was less pronounced and an additional wave could be seen before the anodic copper wave. The additional wave can be explained by the selective oxidation of indium from an alloy containing copper and indium [23]. This explanation was supported by the agreement between the decrease in area of the indium reduction wave and the area of the additional wave. From Figure 2 it could be concluded that indium and gallium in the solution did not influence the potential for electrodeposition of copper.

To mimic a solution after copper separation a solution containing indium(III) and gallium(III) was analyzed. The result was the same as that for a solution containing only indium(III).

**Figure 3:**
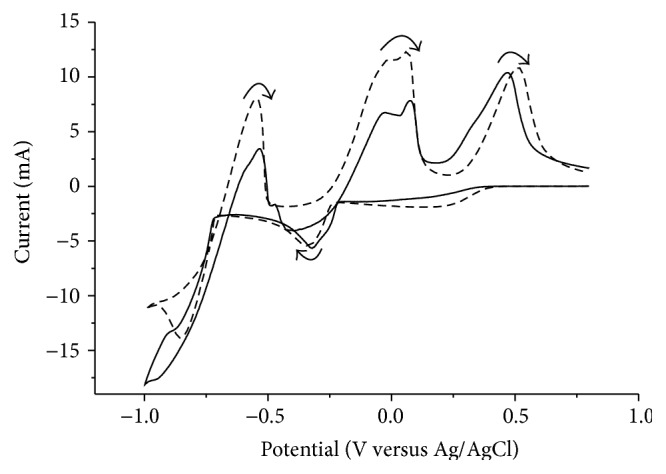
Voltammogram of the two solutions containing dissolved real waste material, Solution 2 (solid line) and Solution 3 (dashed line), at a scan rate of 100 mV/s.

Before the separation investigations, Solutions 2 and 3 (both containing dissolved deselenized CIGS material) were also tested with cyclic voltammetry to determine if the same copper reduction potential as that for the synthetic solutions could be used. The results are presented in Figure 3.

While the voltammogram for Solution 2 was slightly distorted, the voltammogram for Solution 3 was similar to that obtained for the synthetics solution. Both analyses showed that the same copper reduction potential of −0.5 V could be used. After the separation of copper the solutions were analyzed again to determine if the indium reduction potential needed to be modified. The cyclic voltammogram had the same shape as that of the synthetic solution containing only indium (and gallium) and the same indium reduction potential of −0.9 V could therefore be used.

### 3.2. Electrochemical Separation of Copper, Indium, and Gallium from a Synthetic Chloride Solution

**Figure 4:**
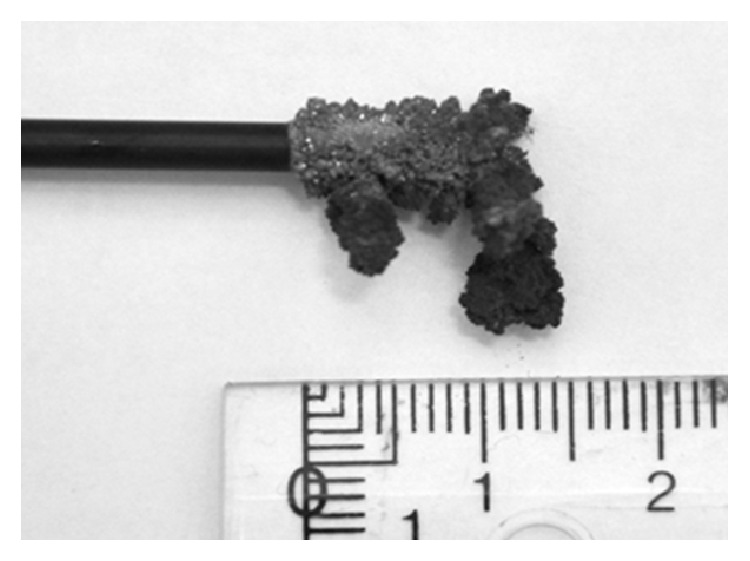
Copper metal from electrodeposition from the synthetic solution (Solution 1), containing copper(II), indium(III), and gallium(III) in 1 M HCl, using a potential of −0.5 V.

The copper deposition formed during 8 h of copper electrodeposition from the synthetic chloride solution (Solution 1) is shown in Figure 4. The deposit had a highly dendritic and powdery morphology, which is typical for electrodeposition of copper from chloride solutions [24, 25].

**Figure 5:**
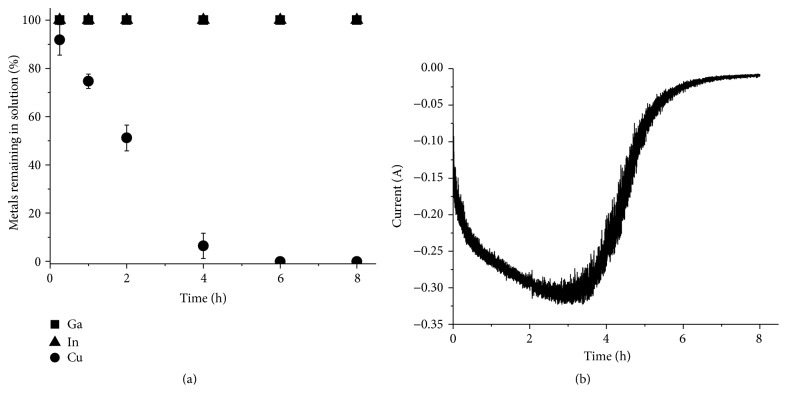
Electrochemical separation of copper from Solution 1. (a) The metal concentration in the solution relative to the original concentration as a function of time. (b) The current passed through the circuit as a function of time.

The concentrations of metals in the solution and the current during the deposition are shown in Figure 5. The increase in cathodic current during the first 3 h is due to the increase in electrode surface due to the dendritic nature of the copper metal. After approximately 5.5 h the solution lost its blue color and ICP-OES analysis (Figure 5(a)) after 6 h showed no significant traces of copper in the solution. The current (Figure 5(b)) was basically 0 after 6 h. The copper yield was determined to be 99.8 ± 0.3% and the indium and gallium concentrations in the copper deposit were below the detection limit of the ICP-OES. From the chronoamperogram (Figure 5(b)) the charge passed during electrodeposition (*Q*) was determined by calculating the peak area using the trapezoidal rule. The current efficiency was determined by comparing the value with the corresponding value calculated using (6).

(6) $$\begin{array}{c} Q=F\cdot N\cdot z, \end{array}$$

where *F* is Faraday's constant, *N* is the number of moles of metal formed, and *z* is the number of electrons transferred per ion. For Solution 1 the current efficiency was found to be 46.3 ± 2.0%.

**Figure 6:**
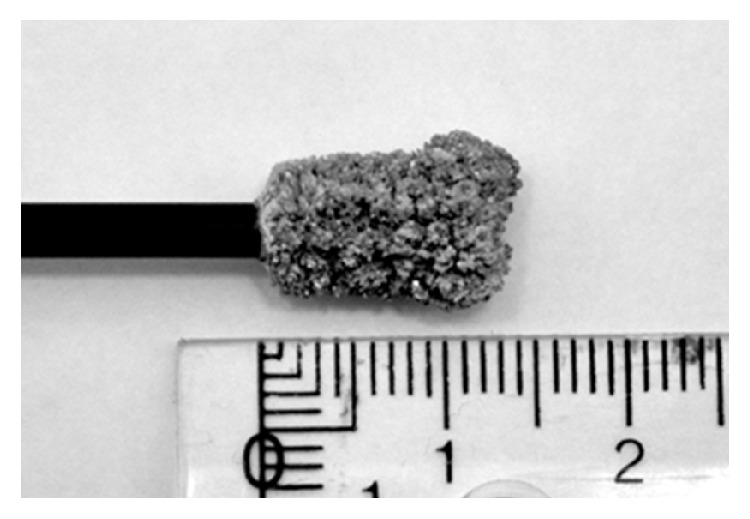
Indium metal from electrodeposition from the copper-depleted synthetic solution after copper separation using a potential of −0.9 V.

Electrodeposition of indium from the copper-depleted synthetic solution for 8 h resulted in the indium metal seen in Figure 6.

**Figure 7:**
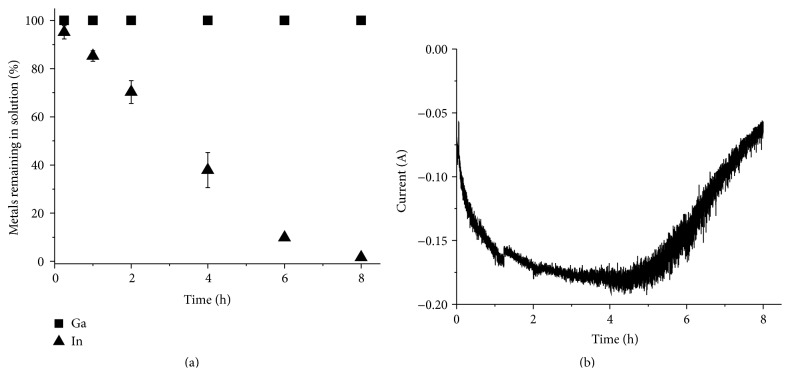
Electrochemical separation of indium from the synthetic solution containing copper(II), indium(III), and gallium(III) in 1 M HCl (Solution 1). (a) The metal concentration in the solution relative to the original concentration as a function of time. (b) The current passed though the circuit as a function of time.

Analysis of the metal concentrations in the solution during the experiment, presented in Figure 7(a), showed that no gallium was removed from the solution, while the indium concentration decreased steadily. After 8 h the indium concentration had decreased to 1.6 ± 0.2% of the original concentration and the indium yield was determined to be 98.4 ± 0.2%. ICP-OES analysis of the indium deposit showed no traces of gallium and it was concluded that a pure indium fraction had been achieved. The current efficiency for the indium separation was calculated in the same way as for the copper separation (Figure 5) and was determined to be 65.9 ± 7.2%.

Thus, the study of the synthetic solution showed that separation of pure copper and indium could be achieved from chloride solutions. It was also clear that the current was higher during the electrodeposition of copper and the time needed for complete separation was therefore shorter for copper than for indium. The area of the copper deposit seems to be larger than the area of the indium deposit and this could be the reason for the higher rate of reduction. In addition, the electrodeposition of copper is catalyzed by the presence of chloride ions in the solution [20, 26]. The reaction rate of the stepwise reduction of copper(II) via copper(I), described above, is higher than the direct reduction to copper(II). However, a similar catalyzing effect has been indicated for indium [27]. Another reason could be that copper ions, due to a smaller ionic radius, diffuse faster towards the electrode than indium ions, but stirring of the solution should practically reduce this difference. The gallium remained in the solution fully separated from copper but with a small contamination of indium.

### 3.3. Electrochemical Separation of Copper, Indium, and Gallium from a Solution of Selenium-Depleted CIGS Waste Material Dissolved in 3 M Hydrochloric Acid and Hydrogen Peroxide

**Figure 8:**
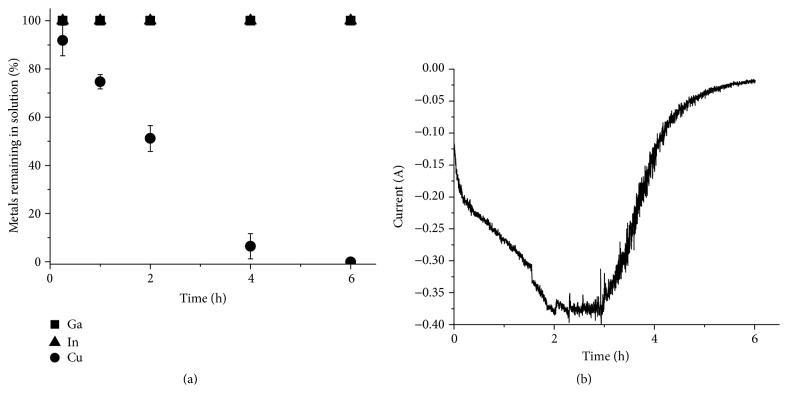
Electrochemical separation of copper from a solution prepared by directly dissolving real waste material in 3 M HCl with an addition of H_2_O_2_ at 80°C (Solution 2). (a) The metal concentration in the solution relative to the original concentration as a function of time. (b) The current passed though the circuit as a function of time.

After the successful separation of copper and indium from the gallium in the synthetic solution, separation of copper was tested on Solution 2, which was prepared by dissolution of real waste CIGS material in 3 M hydrochloric acid with an addition of H_2_O_2_ at 80°C. The results from the separation tests are shown in Figure 8. Since the current was almost 0 A after 6 h it was determined that the experiment could be ended after 6 h. After the separation ICP-OES analysis showed no traces of copper in the solution, while the indium and gallium concentrations in the solution remained constant. The selenium content in the solution also remained constant even though selenium should be codeposited with copper [8, 28]. The lack of selenium reduction might be due to a high stability of the selenium chloride complexes in the solution. The copper yield was 100.0 ± 0.2% and the concentrations of the other elements in the deposit were below the detection limit of ICP-OES. The current efficiency was determined to be 51.5 ± 3.3%.

**Figure 9:**
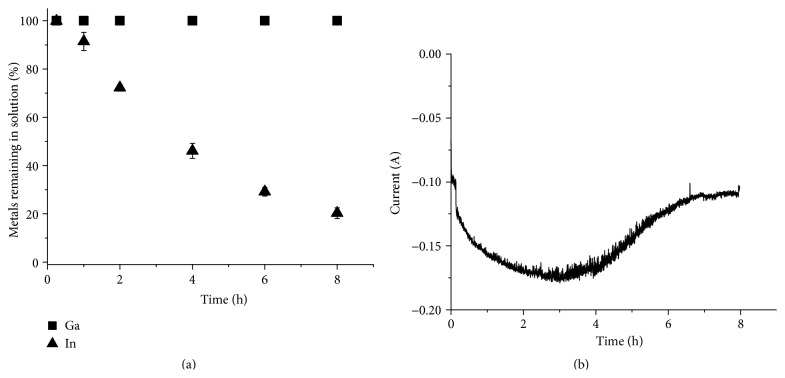
Electrochemical separation of indium from a solution prepared by directly dissolving real waste material in 3 M HCl with an addition of H_2_O_2_ at 80°C (Solution 2). (a) The metal concentration in the solution relative to the original concentration as a function of time. (b) The current passed though the circuit as a function of time.

Next, separation of indium was performed from the copper-depleted Solution 2. As can be seen in Figure 9 the indium concentration decreased during the experiment, while the gallium concentration remained constant. However, 20.3 ± 2.2% of the indium remained in the solution after 8 h and the indium yield was only 76.7 ± 4.7%. It can also be seen that the indium concentration did not decrease linearly and that the current leveled out at approximately 110 mA after 6 h (see Figure 9(b)). During the experiment an increase in gas production at the working electrode was observed. The incomplete indium separation and the gas production could be explained by an increase in proton reduction as the indium concentration decreases, which in turn leads to a lower indium reduction efficiency [29, 30]. Complete separation of indium from the solution might therefore be difficult even using longer time periods. The indium recovery from Solution 2 was lower compared to the indium recovery from Solution 1. The pH levels of the solutions were relatively similar and could not explain the difference. Solution 2 could however still contain hydrogen peroxide from the dissolution, which may influence the reduction of indium. The current efficiency for the indium separation was 68.9 ± 2.5%.

### 3.4. Electrochemical Separation of Copper, Indium, and Gallium from a Solution of Chlorinated, Selenium-Depleted CIGS Waste Material Dissolved in 1 M Hydrochloric Acid

**Figure 10:**
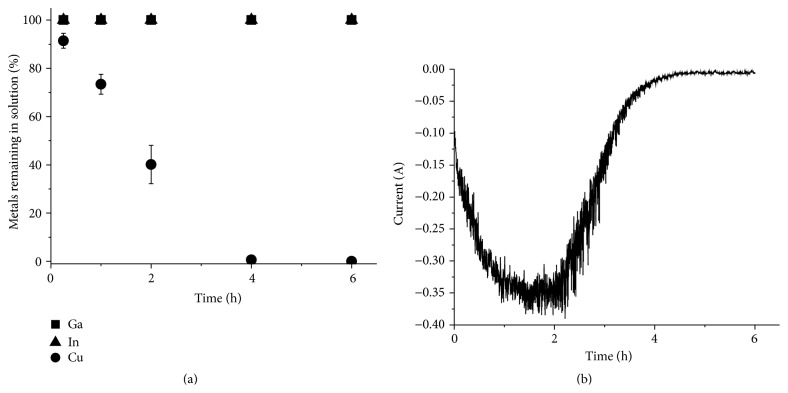
Electrochemical separation of copper from a solution prepared by dissolving prechlorinated CIGS waste material in 1 M HCl (Solution 3). (a) The metal concentration in the solution relative to the original concentration as a function of time. (b) The current passed though the circuit as a function of time.

The result from the separation of copper from Solution 3 is shown in Figure 10. Also in this case the indium and gallium concentrations in the solution remained constant, while the copper content decreased to below 0.5% in 4 h. The copper yield after 6 h was 100.1 ± 0.5%. The indium and gallium content in the deposit were below the detection limit for the ICP-OES and the current efficiency was 68.5 ± 2.5%.

**Figure 11:**
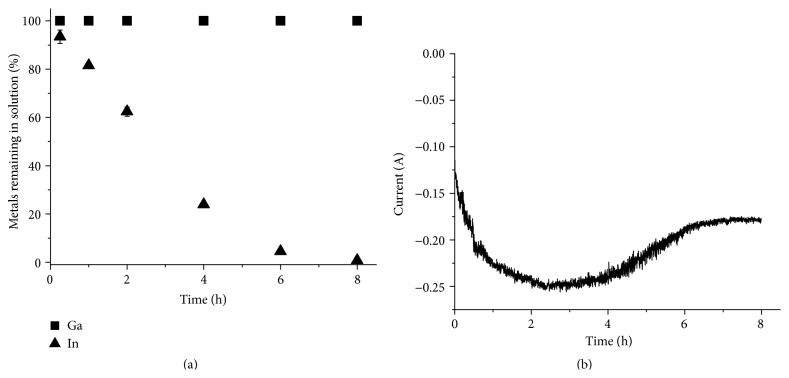
Electrochemical separation of indium from a solution prepared by dissolving prechlorinated CIGS waste material in 1 M HCl (Solution 3). (a) The metal concentration in the solution relative to the original concentration as a function of time. (b) The current passed though the circuit as a function of time.

Finally, indium was separated from the copper-depleted Solution 3 (Figure 11). Less than 1% of the indium remained in the solution after 8 h of electrodeposition and the indium yield was 98.1 ± 2.5%. The current efficiency for the indium separation was determined to be 61.2 ± 1.7%. Similarly to the indium separation from Solution 2, the current leveled out after 6 h and an increase in hydrogen gas production at the working electrode could be seen as the indium concentration in the solution decreased. However, in this case the indium separation was still close to complete and the solution contained less than 1% of the original amount of indium. It has been reported that ammonium chloride addition can be used to get chloride solutions with higher conductivity [31]. One explanation for the difference in indium separation could be that the conductivity of the ammonium chloride containing solution (Solution 3) is higher than the conductivity of the hydrogen chloride solution (Solution 2), leading to a higher current and a higher indium reduction rate.

All gallium remained in the solution after separation of pure copper and indium fractions as in previous experiment series. The solution also contained some indium, but no measureable amounts of copper or selenium. The ratio between gallium and indium in the solution was 97.4 ± 5.2 mole% gallium to 2.6 ± 0.1 mole% indium.

### 3.5. Comparison of the Morphology of the Deposits from Electrodeposition of Copper and Indium from the Different Solutions

The morphology of the copper deposits from Solutions 2 and 3 was slightly more powdery than the deposit from Solution 1. It has been shown that higher current densities give rough and powdery deposits [24, 25] and if Figures 8 and 10 are compared with Figure 5 it can be seen that the current was significantly higher during electrodeposition from Solutions 2 and 3 than that from Solution 1. In order to get smooth and dense deposits the current density, the stirring rate, the copper and chloride concentrations in the electrolyte, and temperature could be modified [24]. In addition nitrogen sparging and additions of bone glue could be used to produce more dense and smooth deposits [25]. The weight of the copper deposits formed during electrodeposition from all three solutions in the present study corresponded well with the amount of copper measured with ICP-OES. Since no additional material was present in the deposit it is most likely that no CuCl contamination, which can be a problem in chloride solutions [24, 25], had been formed.

The deposits formed during the separation of indium from Solutions 2 and 3 had similar, dendritic structures to the one from the synthetic solution (see Figure 6). To decrease the roughness of the deposits the current density, indium concentration, and additives, such as glue, thiourea, and sodium lignin sulfonate, have to be carefully selected [31, 32].

### 3.6. Comparison of the Current Efficiencies for Electrodeposition of Copper and Indium from the Different Solutions

According to Kekesi and Isshiki, the current efficiency for copper electrodeposition from chloride solutions is influenced negatively by a high hydrochloric acid concentration, since the formed copper is partially redissolved. A high current density in a constant current experiment will, on the other hand, give a higher current efficiency since the redissolution is counteracted [24]. The high chloride concentrations in the three solutions tested in this study could explain the low current efficiencies (46.3 ± 2.0% for Solution 1, 51.5 ± 3.3% for Solution 2, and 68.5 ± 2.5% for Solution 3). The current efficiencies were probably also lowered by particles released from the working electrode during the experiments. The majority of the particles seemed to be redissolved and no particles could be seen in the solution at the end of the experiment. The ammonium chloride containing solution (Solution 3) gave the highest current efficiency for the copper separation and further optimization of the ammonium chloride and hydrochloric acid concentrations in the solution and the current density might lead to even higher efficiencies.

From experiments on gallium it has been concluded that a larger current density gives a higher metal reduction rate, but the current efficiency is affected negatively [33]. Since indium is similar to gallium this could also be the explanation for the lower current efficiency for the indium separation from Solution 3 (61.2 ± 1.7%) compared to the other solutions (65.9 ± 7.2% and 68.9 ± 2.5%). According to Lee and Sohn, current efficiencies over 90% can be achieved if the indium concentration is above 0.44 M (50 g/L) and at concentrations below 0.26 M (30 g/L) the current efficiency decreases due to proton reduction [29]. However, when the goal is to completely deplete the solution the indium concentration will inevitably be below those concentrations and other means (e.g., optimal current densities) have to be used to increase the current efficiency.

### 3.7. Considerations for Further Development of a Process for Separating the Deselenized Waste CIGS Material

From the copper and indium separation studies it can be concluded that the ammonium chloride containing solution (Solution 3) is the most promising for further development of an electrochemical recycling process for recovering of copper and indium from the selenium-depleted CIGS waste material. It gave both the highest current efficiency for the copper separation, the highest copper reduction rate, and the best indium separation. In this study the potential was kept constant in order to ensure good separation of the elements. In future studies, however, the current density should be kept constant and different current densities should be tested in order to optimize the current efficiencies and to produce smooth and dense deposits. The hydrochloric acid and ammonium chloride concentrations in the solution could also be modified and, if necessary, additives could be used for further improvements.

The difficulty in electrowining gallium from the tested chloride solutions also makes further development of the process necessary. Since electrodeposition of gallium is often done from alkaline solutions [12, 33–41] one option could be to precipitate the gallium as gallium hydroxide and proceed with the electrodeposition after dissolution of the precipitate.

During electrodeposition from the chloride solutions it was observed that chlorine gas was produced at the counter electrode. The chloride in the solution is oxidized to chlorine gas according to the following reaction:

(7) $$\begin{array}{c} 2{Cl}^{-}\left(\;aq\;\right)⟶{Cl}_{2}\left(\;g\;\right)+e^{-} \end{array}$$

This is an unwanted reaction, both because the electrolyte is destroyed and because of the difficulties related to taking care of the gas produced. To avoid chlorine gas production a two-compartment cell, where the anode is separated from the chloride solution by a membrane, could be used [32]. As another solution to the problem it has been suggested that the chlorine gas could be used to regenerate the electrolyte [42]. In our case the chlorine gas could be used instead of ammonium chloride to chlorinate the oxidized waste material. According to previously published work, complete chlorination of the material with chlorine gas is possible at 750°C [18]. This would give a highly water-soluble material and solutions similar to the synthetic solution tested in this study.

## 4. Conclusions

We have investigated the feasibility of separating copper, indium, and gallium into pure fractions from a chloride-rich solution from leaching of pretreated CIGS waste material using an electrochemical method. Suitable reduction potentials for electrodeposition of copper and indium were determined to be −0.5 V and −0.9 V (versus Ag/AgCl), respectively. No reduction of gallium could be detected in the examined potential window, making separation of indium and gallium easier than expected.

Electrochemical separation of first copper and then indium from three different solutions was tested. First a synthetic chloride solution was used to prove that the separation was possible; then two solutions from real selenium-depleted CIGS solar cell waste material were studied. Of the two CIGS solutions the ammonium chloride containing solution gave the best results. Both solutions gave a complete copper separation, but the ammonium chloride containing solution gave the highest current efficiency and the highest reduction rate for the copper separation. It also gave a much higher and almost complete indium separation (98.1 ± 2.5%) compared to the other solution (76.7 ± 4.7%). The separated metals contained no contamination of the other elements, but both the copper and the indium deposits had a highly dendritic morphology. To increase the current efficiencies and produce dense and smooth deposits further studies, where the current density is kept constant instead of the potential, are needed in order to find the optimum current density. The chloride concentration in the solution could very well also be optimized and additives could be used to achieve better results. We also suggest that the chlorine gas produced at the counter electrode could be used, instead of ammonium chloride, to chlorinate the selenium-depleted CIGS waste material.

We have shown that separation of copper, indium, and gallium is possible using electrodeposition from hydrochloric acid media. The difficulty in electrodepositing gallium makes the separation of the elements easier, but development of an efficient method to recover the gallium in pure form is needed.
